# Primary sternal tumour resection and reconstruction with LARS mesh-bone cement sandwich by 3D-printing: Case reports

**DOI:** 10.3389/fbioe.2023.1024480

**Published:** 2023-04-07

**Authors:** He Zhang, Bo Hou, Tienan Xia, Lu Ji, Jiatong Li, Ting Chen, Guanning Shang

**Affiliations:** ^1^ Department of Bone and Soft Tissue Oncology, Department of Surgery, Shengjing Hospital, China Medical University, ShenYang, Liaoning, China; ^2^ Department of Orthopedics, Fifth People’s Hospital of Shenyang, ShenYang, Liaoning, China; ^3^ Department of Gynecology and Obstetrics, Shengjing Hospital, China Medical University, ShenYang, Liaoning, China

**Keywords:** sternal tumour, LARS, reconstruction, bone cement, 3D-printing, case reports

## Abstract

**Background:** There are many reconstruction methods after sternal tumor resection, but the method that LARS mesh combines with bone-cement has not been reported.

**Case report:** A 54-year-old female patient and a 55-year-old male patient admitted to our department all presented with sternum masses, but neither presented with respiratory disorders. In women with limited manubrium sternum lesions, we resected the manubrium sternum completely. In men with sternal lesions, we removed part of the sternum and part of the sternocostal joint. The patients recovered well after surgery, and there were no respiratory complications and no tumor recurrence during the 1-year follow-up respectively.

**Conclusion:** We report two cases of sternal defect repair using LARS mesh combined with bone cement. This method is safe and stable, and can achieve satisfactory results.

## 1 Introduction

Primary chest tumours are infrequent, accounting for less than 1% of primary bone tumours (Chaudhry et al., 2015; Ma et al., 2018), and only 20% of chest tumours arise from the sternum (Hann et al., 2018). Although primary sternal tumours are rare, their prevalence has increased in recent years. Among sternal tumours, malignancies such as chondrosarcoma are the most common. Benign lesions include chondromas, osteomas, and fibrous dysplasia (Gritsiuta et al., 2021). Afflicted patients often complain of a palpable chest mass accompanied by pain or cough.

Surgery is the primary treatment for sternal tumours when obvious clinical symptoms occur, whether malignant or benign. Radical resection is the optimal therapy for surgeons to guarantee non-tumour margins and low recurrence rates (Zhang et al., 2015). However, a larger excision area indicates a larger chest defect. The chest wall plays a critical role in thoracic stability and respiratory function. Reconstruction of chest defects should be considered to protect thoracic organs, restore cosmetic appearance, and prevent paradoxical ventilatory function (Zhang et al., 2015).

Reconstruction usually consists of two aspects: skeletal reconstruction and soft tissue reconstruction (Khullar and Fernandez, 2017). Soft tissue defects are usually resolved using primary closure or myocutaneous flaps, such as the latissimus dorsi, pectoralis major, serratus anterior, and rectus abdominis muscles. The optimal materials used in skeletal reconstruction are currently under debate, and no consensus has been reached. Such materials should have the properties of rigidity, biocompatibility, flexibility, and elasticity. Historically, various prosthetic materials used in reconstruction have included synthetic meshes, bioprosthetic materials, stainless steel, titanium plates, autografts, and homografts (Sandler and Hayes-Jordan, 2018). Each technique has its advantages and disadvantages. The choice of reconstruction method depends on the patient’s state, the surgeon’s preference, and the availability of materials.

In this cases, we support the use of an innovative ligament advanced reinforcement system (LARS)-bone cement sandwich as a prosthesis for reconstruction of the chest wall, which has not yet been reported in the literature. This new method achieved a good therapeutic effect, with uneventful recovery at the 1-year follow-up.

## 2 Case reports

### 2.1 Demographics

**FIGURE 1:**
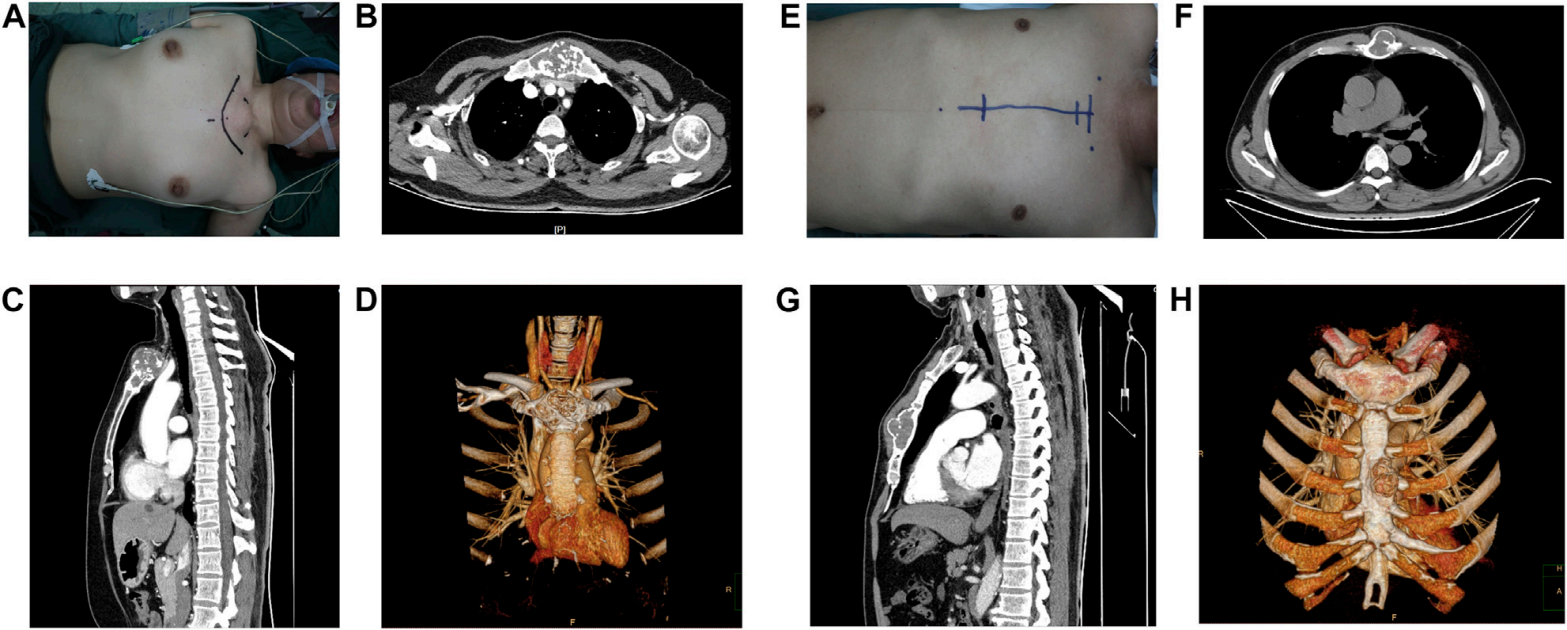
Appearance and preoperative images of the patients. **(A–D)** Female patient and **(E–H)** Male patient. **(A,E)** Appearance of the lesion and operative route on the body surface. **(B,C) (F,G)** The tumour is shown in cross-sectional and sagittal views **(D,H**), **(D)** reconstruction of tumour on CT is demonstrated.

We report the cases of a 54-year-old female patient and a 55-year-old male patient who presented to our department with sternal masses, from March to May 2021; both patients were operated on by the same doctor. In the female patient, the lesion was located in the manubrium sternum, and was diagnosed as chondrosarcoma by postoperative pathology. In the male patient, the lesion was located in the middle of the sternum with involvement of the fourth rib, and was diagnosed as bone fibrous dysplasia based on postoperative pathology (Supplementary Table S1, Supplementary Figure S1). Both patients underwent preoperative chest 3-dimention computed tomography (slice thick 1 mm, 120 kV, 346 mA, FOV 500 mm) (Figure 1). Meanwhile, electrocardiography, pulmonary function tests, and other necessary examinations were conducted with normal results to ensure the surgery safety. Both patients denied other diseases. We made a 3D printed model based on the computed tomography data so as to determine the lesion and excision scopes, and to design the bone cement mould for surgery. 3D printing technology was achieved by mimics software, 3-matic software and 3D printer (RAISE 3D-n2, Shanghai Fuzhi Technology Co., LTD.). We imported the CT data into the mimics software to design 3D model according to the surgical requirement. Next, The modeled 3D data was imported to 3D-printer to manufacture 3D model using the polylatex (PLA). Serological examinations, including blood tests, liver and kidney function, and serum ions, were normal.

### 2.2 The sternum defect was repaired with polymethyl methacrylate (PMMA) and LARS ligament

#### 2.2.1 Anaesthetic consideration

Both patients were placed in the supine position during the operation. The patients were anaesthetised using two-chamber endotracheal intubation after routine testing of blood oxygen and blood pressure and evaluating the electrocardiogram findings. The arterial catheter was indwelling for observation of blood gas analysis in surgery.

#### 2.2.2 Resection

For sternal tumours, we ensured an adequate resection area according to individual principles, which meant the confirmation of incision and excision margin depended on intrinsic properties of tumours. Preoperative printing of the 3D model played a positive role in understanding the lesion scope and determining the surgical plan. For the female patient with a lesion limited to the manubrium sternum, the platysma muscle, sternocleidomastoid muscle, sternoclavicular joint, sternocostal joint, and the joint between the sternocleidomastoid and sternum were sequentially severed after exposure. For the male patient with a lesion located in the sternal body, we separated the sternal body from the manubrium sternum and bilateral third-sixth sternal costal joints. The residual costal stumps were proven to be non-tumourous on intraoperative pathology examination. The tumor histological characteristics of the female patient presented as lobulated hyaluronic cartilage nodules, which resembled normal cartilage to some extent. The margin of the tumor was white and yellow granular. The tumor histological characteristics of the male patient presented as no obvious boundary of osteocele, cystic fibrous changes, and the bone marrow cavity was occupied by grayish white or grayish red hyperplastic fibrous tissue.

#### 2.2.3 Bone cement prosthesis and LARS ligament coverage

**FIGURE 2:**
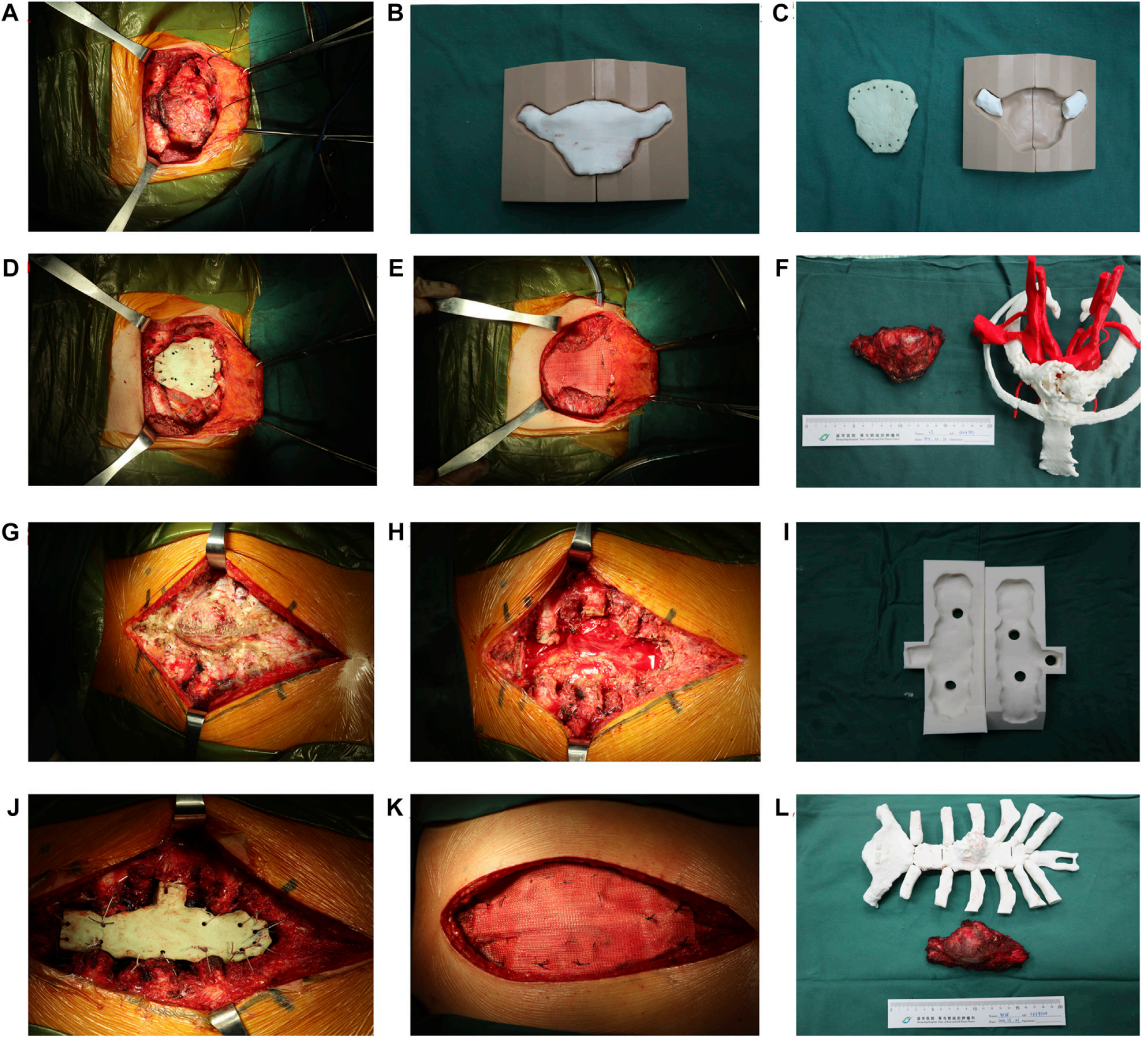
Surgical procedure of sternal tumour resection and reconstruction. **(A–F)** The female patient. **(A)** The lesion was exposed and separated. **(B–C)** Fabrication of sternal prosthesis by 3D-printing. **(D–E)** A “Sandwich” cement prosthesis is implanted in chest defect region. **(F)** The resected specimen and bone cement prosthesis were compared with the 3D-printing model. **(G–L)** The male patient. **(G–H)** The lesion is exposed and removed. **(I)** Fabrication of the sternal prosthesis by the 3D-printing mould. **(J–K)** A “Sandwich” cement prosthesis is implanted in the chest defect region. **(L)** The resected specimen and bone cement prosthesis as compared to the 3D-printing model.

After removing the sternal lesions, bone cement was poured into a pre-made mould. The mould was fabricated using 3D printing technology based on CT information to ensure its suitability. This mould is generally composed of two parts: the bottom and the cover. During fabrication, we first poured an appropriate amount of paste bone cement into the bottom and then covered it with a cover to ensure that the prosthesis surface was smooth. Subsequently, holes were drilled at the edge of the bone-cement prosthesis. The bone cement prosthesis was implanted in the sternal defect, and it was firmly fixed on the adjacent ribs and the residual sternum with non-absorbable wires. Next, the sternocleidomastoid muscle was sutured to the cement, which stabilised the sternocleidomastoid joint. We then fixed the LARS mesh to the surface of the cement prosthesis and the peripheral ligament on the pectoralis major muscles through interrupted non-absorbable sutures on the holes reserved in the cement. A drainage tube was inserted in both the patients during the operation. The surgical procedures were seen in Figure 2.

#### 2.2.4 Postoperative care

Both patients were routinely transferred to the ward for primary care after surgery. They were provided with postoperative anti-inflammatory and analgesic therapy, and their respiratory function was closely monitored. When the drainage flow was less than 20 mL for 2 days, the drainage tube was removed. The average drainage time of the two patients was 8.5 days, and the average hospital stay was 16.5 days. No major postoperative complications occurred in either patient. The male patient experienced mild dyspnea on the night of surgery, which may have been caused by incision pain.

**FIGURE 3:**
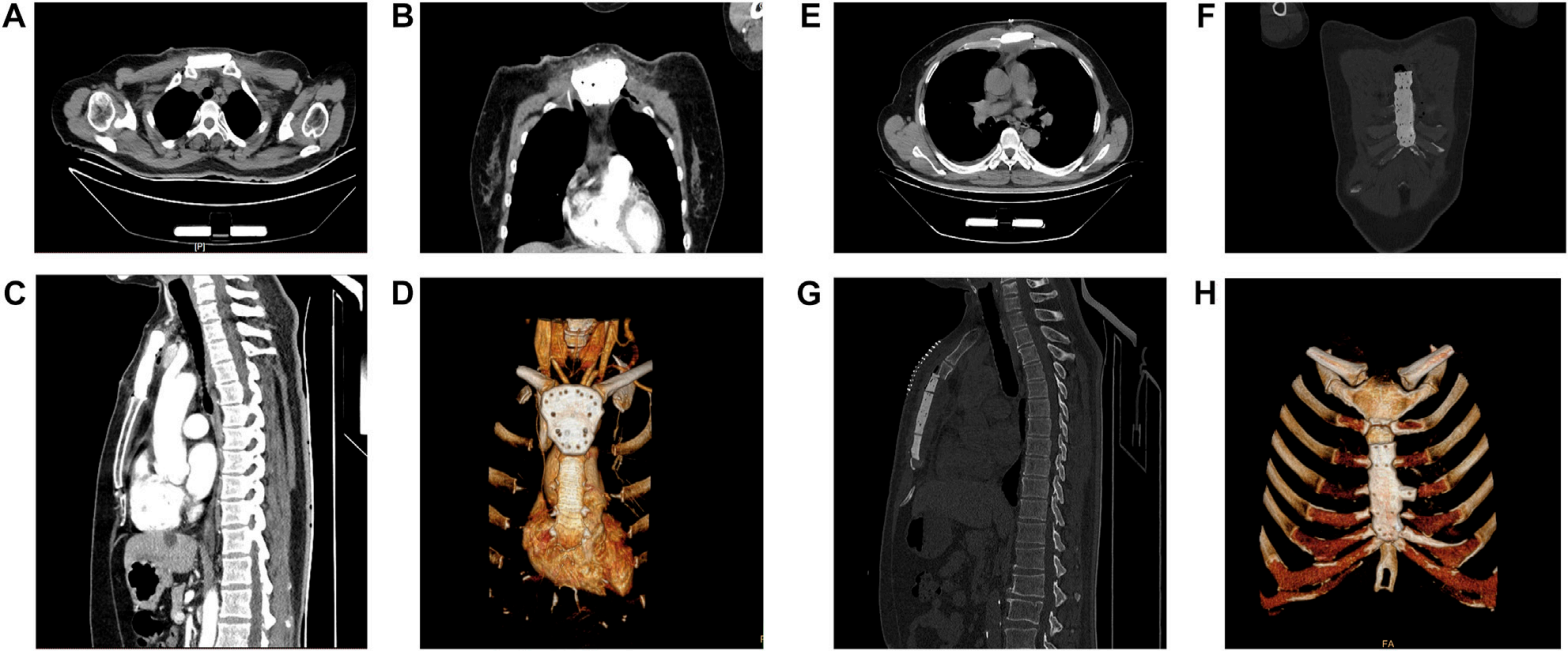
Postoperative computerized tomography (CT) images of the patients. **(A,E)** The CT images of sternal prosthesis in the transverse view.**(B,F)** The CT images of sternal prosthesis in the coronal view. **(C,G)** The CT images of sternal prosthesis in the sagittal view. **(D,H)** The 3D reconstruction of the sternal prosthesis on CT is demonstrated.

**FIGURE 4:**
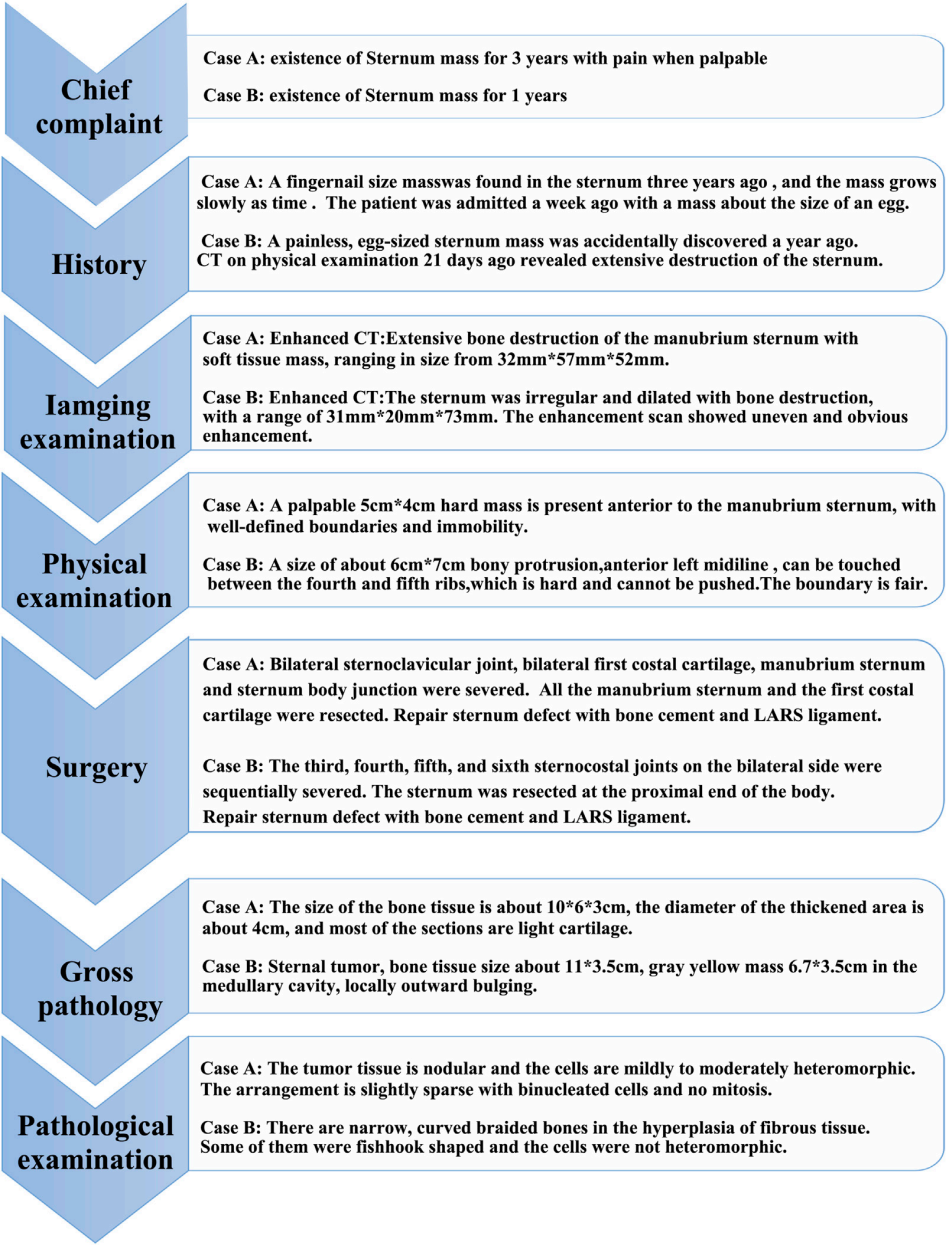
The therapeutic course of our patient. The current case series is presented based on this sequence of events.

Postoperative pain management is extremely important in patients undergoing sternal resection. Furthermore, thoracic movement should be limited to avoid implant displacement. A small range of implant forward movements could be accepted, while implant back movements must be avoided, as this could lead to respiratory and circulatory failure. Both patients underwent chest tomography after drainage extubation that revealed sternal prosthesis located at proper position and cover the defect well (Figure 3). A follow-up 1 year postoperatively showed satisfactory cosmetic results and good self-assessment without any abnormal respiratory movements or dislocation of the prosthesis in either patient. The therapeutic procedure of this case report was seen in Figure 4.

## 3 Discussion

The sternum is a rare site for neoplasms, and surgery is the most important treatment for sternal tumours (Ersoz et al., 2014). Considering the role of the chest wall in respiratory function, protection of thoracic organs, and cosmetic appearance, reconstruction of large chest defects has reached a consensus, although the choice of material for chest reconstruction is still controversial (Wang et al., 2020).

It has been reported that various materials were used in chest reconstruction. Rigidity is the most important property that can prevent flail chest and protect organs (Nishida et al., 2015). In this case, the bone cement, titanium plates, and autografts could meet the requirements for rigidity. The autograft has rigidity with good self-compatibility, which makes it both resistant to infection and implantable (Drinnon et al., 2020). However, the corresponding incision exerts a significant pain burden on patients (Huang et al., 2015). Titanium plates have become a sophisticated material in recent years. The light weight, strength, durability, biocompatibility, and non-magnetic properties of titanium make it a preferable material for implants (Goldsmith et al., 2020). Nevertheless, there are still some limitations to the use of titanium plates in reconstruction. The lack of elasticity and tenacity of the titanium plates may increase the incidence of post-operative prosthetic dislocation (Voss et al., 2018). Meanwhile, titanium plates are often secured with screws on the ribs, which can result in unscrewing of the screws and dislocation of the prosthesis owing to the osteoporotic nature of the ribs (Ng, 2015). Furthermore, the high price of titanium may also be a considerable limitation (Divisi et al., 2021). Although the most common complication of PMMA is infection, LARS mesh could promote the adhesion and development of soft tissues, which could decrease risk of infection.

To overcome these bottlenecks, we proposed a new sandwich structure, referred to as PMMA, encapsulated with a LARS mesh. PMMA can provide sufficient support to the chest wall (Motono et al., 2019), is easy to manipulate, and less time-consuming to mould into sternal prostheses, due to the potential of a 3D-printing precast mould and the high plasticity of PMMA in the early period (Choi et al., 2021). In this case, we secured the prosthesis to the ribs with interrupted 1–0 sutures to reduce the incidence of prosthesis displacement caused by the osteoporotic nature of the ribs.

In addition to PMMA, the LARS mesh is also essential for chest reconstruction. The restoration of respiration and movement of the upper limbs required micro-motion of the sterno-clavicular sternocostal joints, which was possible due to the elastic property of the mesh (Chaudhry et al., 2015). Traditional meshes, such as Marlex mesh, Prolene mesh, and Gore-Tex, are commonly used in clinics (Sandler and Hayes-Jordan, 2018; Motono et al., 2019; Tsuge et al., 2021). There was also a limitation of the technology. The absorption and shrinkage of traditional meshes can result in chest pain and wall deformities. Several studies have revealed a shrinkage range of bio-meshes from 7.6% to 50.8%, which was caused by degradation of the absorbable element of the mesh over time (Huang et al., 2015). Considering the limitations of traditional meshes, the LARS mesh was applied to wrap the PMMA prosthesis during surgery because of its remarkable strength, durability, elasticity, and bio-stability (Tiefenboeck et al., 2018; Naveen et al., 2020); this combination prevented absorption and shrinkage. The microporous structure of LARS also provides a scaffold that facilitates the adhesion of soft tissues (Trieb et al., 2004). Patients with LARS-PMMA sandwich prostheses had restored chest and shoulder strength, allowing normal respiration and movement of the upper limb. Although our treatment protocol has achieved satisfactory results, there are also some shortcomings as follows: 1) the particulate matter produced by the abrasion of the LARS ligament may cause an inflammatory reaction and its longevity may be shortened by long-term respiratory motion (Wei et al., 2019); 2) The bone cement may reduce the range of motion of the joint and may inhibit respiratory activity, and all synthetic materials remain permanently in the body after implantation, and there is also a risk of dislocation and deflection of the implanted prothesis (Chaudhry et al., 2015). Moreover, the loosening and fracture of the prosthesis may occur with prolonged implantation leading to chest wall deformity or even respiratory contradiction, although the probability is very low.

Besides chest wall reconstruction, soft tissue coverage of the prosthesis is necessary to prevent infection. In different situations, primary closure, skin grafts, myocutaneous flaps, and local advancement flaps may be used (Hameed et al., 2008). In this case, we covered the sternal prosthesis through a primary suture based on sufficient residual soft tissue.

## 4 Conclusion

Sternal tumours are rare, and are generally treated by tumour resection followed by chest defect reconstruction, which remains a challenging procedure. Bony and soft tissue reconstructions are two prominent aspects of maintaining chest cosmesis, respiratory function, and organ protection. The LARS-PMMA-LARS sandwich prosthesis has potential to be a suitable substitute for the sternum. Proper soft tissue reconstruction also promotes restoration of chest function and structure.

## Data Availability

The original contributions presented in the study are included in the article/Supplementary Material, further inquiries can be directed to the corresponding authors.
